# Preoperative Upper-GI Endoscopy Prior to Bariatric Surgery: Essential or Optional?

**DOI:** 10.1007/s11695-020-04485-5

**Published:** 2020-02-24

**Authors:** Yusef Moulla, Orestis Lyros, Matthias Mehdorn, Undine Lange, Haitham Hamade, Rene Thieme, Albrecht Hoffmeister, Jürgen Feisthammel, Matthias Blüher, Boris Jansen-Winkeln, Ines Gockel, Arne Dietrich

**Affiliations:** 1 Department of Abdominal, Transplant, Thoracic and Vascular Surgery, University Hospital of Leipzig, Liebigstrasse 20, D-04103 Leipzig, Germany; 2 Integrated Research and Treatment Center (IFB) Adiposity Diseases, Leipzig, Germany; 3 Clinic for Gastroenterology, Department of Internal Medicine, University Hospital of Leipzig, Liebigstrasse 20, D-04103 Leipzig, Germany

**Keywords:** Preoperative endoscopy, Perioperative management, GERD, Barrett’s esophagus, Bariatric surgery

## Abstract

**Introduction:**

The role of preoperative upper-gastrointestinal (GI) gastroscopy has been discussed with controversy in bariatric surgery. The aim of this study was to evaluate the incidence of upper-GI pathologies detected via endoscopy prior to bariatric surgery along with their clinical significance for patients’ management.

**Material and Methods:**

In our single center prospectively established database of obese patients, who underwent bariatric surgery from January 2011 to December 2017, we retrospectively analyzed the perioperative endoscopic findings along with their influence on patients’ management.

**Results:**

In total, 636 obese patients with median BMI (body mass index) of 49 kg/m^2^ [range 31–92] received an upper-GI endoscopy prior to bariatric surgery. Among the surgical procedures, laparoscopic Roux-Y-gastric bypass (72.6%; *n* = 462) was the most frequent operation. Endoscopically detected pathological conditions were peptic ulcer 3.5% (22/636), Helicobacter pylori (Hp) gastritis 22.4% (143/636), and gastric or duodenal polyps 6.8% (43/636). Reflux esophagitis could be detected in 139/636 patients (21.9%). Barrett’s esophagus (BE) was histologically diagnosed in 95 cases (15.0%), whereas BE was suspected endoscopically in 75 cases (11.3%) only. Esophageal adenocarcinomas were detected in 3 cases (0.5%). Change of the operative strategy due to endoscopically or histologically detected pathologic findings had to be performed in 10 cases (1.6%).

**Conclusion:**

Preoperative upper-GI endoscopy identifies a wide range of abnormal endoscopic findings in obese patients, which may have a significant impact on decision-making, particularly regarding the most suitable bariatric procedure and the appropriate follow-up. Therefore, preoperative upper-GI endoscopy should be considered in all obese patients prior to bariatric procedure.

## Introduction

The incidence of morbid obesity has rapidly been increasing in the last decades along with the number of bariatric procedures worldwide simultaneously. Currently, bariatric surgery is considered the most effective therapy for morbid obesity when dietary and exercise therapies have failed [1]. Apart from the surgical expertise, an appropriate preoperative patients’ workup is essential for a safe and effective bariatric outcome. The accurate preoperative evaluation of bariatric patients, including upper-GI endoscopy aims to facilitate the selection of the best and most appropriate bariatric procedure in order to gain the best weight loss and to improve the obesity associated diseases without however increasing the early and the late morbidity and mortality.

Morbid obesity has been reported as a risk factor for multiple diseases in the upper-GI tract, such as gastritis, hiatal hernia, consecutive gastroesophageal reflux disease (GERD), and Barrett’s esophagus (BE) [2, 3]. Already several reports have assessed the range of the various abnormalities in the upper-GI tract in obese patients [4, 5] and emphasized the necessity of preoperative upper-GI endoscopy. Thus, the current German guidelines recommend that all bariatric patients should undergo upper-GI endoscopy [6]. On the other hand, the American Society for Metabolic & Bariatric Surgery (ASMBS) guidelines have recommended that the indication to perform upper-GI endoscopy in bariatric surgery should be individualized [7]. However, the value of endoscopic findings is still unclear and a matter of controversy within the bariatric community. Especially, the cost-effectiveness of the examination has been questioned, when the majority of the findings do not significantly influence the bariatric course.

The aim of this study was to assess the prevalence of disease-related findings in the upper-GI tract in our cohort of bariatric patients and to examine the clinical relevance of the endoscopic findings for the surgical and postoperative course of the bariatric patients.

## Patients and Methods

### Study Population

We retrospectively analyzed all patients who underwent upper-GI endoscopy prior to bariatric surgery in our center between January 2011 and December 2017. Patients with missing endoscopic or pathological reports were excluded (*n* = 20; 3%). All patients underwent a standardized upper-GI endoscopy with a flexible endoscope by highly experienced endoscopists or supervised residents. During endoscopy, the esophagus, whole stomach, and the descending part of the duodenum were thoroughly inspected following a standardized protocol. The closure of cardia and the presence of hiatal hernia were evaluated by retrograde sight and insufflation. Esophageal biopsies were done mainly in patients with signs of reflux, suspicion of BE, or in the case of Z-line irregularity. Gastric biopsies were routinely taken from the antrum and corpus. Other pathological findings in the upper-GI tract such as polyps or tumors were also biopsied routinely. The presence of Helicobacter pylori (Hp) was assured through the rapid urease test (RUT) and histological staining (silver staining) in patients with gastritis. Esophagitis was classified according to the Savary-Miller classification [8] and gastritis according to updated Sydney system [9]. Patients were divided into 3 groups (Table 1): (a) group 1, no change of the perioperative strategy; (b) group 2, change of the perioperative strategy; and (c) group 3, change of the final operative strategy. Patients with Hp gastritis were offered treatment for Hp without any routine confirmation of eradication postoperatively. Re-endoscopy after eradication and high doses of proton-pump inhibitors (PPIs) were recommended in patients with gastric ulcer or severe hemorrhagic gastritis and Hp infection. Generally, we planned a gastric bypass in patients with gastroesophageal reflux disease (GERD) and BE. In case of BE, we recommended a re-endoscopy after one and then 3 years to re-evaluate the Barrett’s mucosa (according to the current German guidelines) [10]. Standard bariatric procedures were laparoscopic Roux-Y-gastric bypass (LRYGB) and laparoscopic sleeve gastrectomy (LSG) in our clinic. The majority of re-do operations were LSG to LRYGB. Reasons to perform re-do surgery were mainly GERD or weight regain.

**Table 1:** Patients’ groups of changing the operative strategy according to endoscopic findings

*Group*	*Endoscopic findings*	*Clinical relevance*
Group I	No relevant endoscopic or histological findings	No change of the perioperative and operative strategy
Group II	Hp infection, BE, gastric ulcer, severe hemorrhagic gastritis	Change of the perioperative strategy
Group III	Autoimmune gastritis, malignancies, GERD, and BE	Change of the operative strategy

*Hp* Helicobacter pylori; *GERD* gastroesophageal reflux disease; *BE* Barrett’s Esophagus

In all cases of surgery, the esophageal hiatus (left crus) and angle of His was dissected and fully inspected intraoperatively in order to detect or exclude the presence of a hiatal hernia. All detected hiatal hernia were closed intraoperatively.

Furthermore, factors such as age, gender, BMI,^1^ weight, diabetes mellitus, clinically apparent GERD, and obstructive sleep apnea syndrome (OSAS) were analyzed with regard on their effect on the pathological esophageal findings (esophagitis, BE).

## Statistical Analysis

Categorical data were expressed as absolute or relative frequencies. Continuous data were expressed as median and interquartile range or mean and standard deviation. In the univariate analysis, Pearson’s Chi-square and, if necessary, Fisher’s exact test were used to compare proportions with categorical variables and independent sample *t*-test, as well as Mann-Whitney *U* test, to evaluate these with continuous/discrete variables according to the normality distribution depending on Shapiro-Wilk test. Patient’s variables with *P* value less than 0.1 were entered stepwise into a multivariate binary logistic regression to identify the predictors of abnormal upper gastrointestinal endoscopy (UGE) (especially GERD and BE). Significance level was chosen 0.05 and all tests were 2-sided. We used SPSS (v. 20.0) for Windows 10 for all statistical analyses.

## Results

### Patient’s Characteristics

We included 636 patients in our analysis with a median age of 49 years [range 13–75 years] and median body mass index (BMI) of 49 kg/m^2^ [range 31–92 kg/m^2^]. There were 214 (33.6%) males and 422 (66.4%) females. The main bariatric procedure was LRYGB (72.6%; *n* = 462). LSG was performed in 128 patients (20.1%), and re-do operations such as LSG to LRYGB, LSG to single-anastomosis duodenoileal bypass with sleeve gastrectomy SADI-S operation, and gastric banding to LRYGB were performed in 42 patients (6.6%). Hiatal hernia could be detected intraoperatively in 96 patients (15.1%), and subsequently hiatal hernia repair was carried out. LSG was converted into another bariatric procedure in 34 patients (5.3%). However, GERD was displayed in 20 patients of these (58.8%; *n* = 34). The pre-endoscopic bariatric co-morbidities are summarized in Table 2*.*

**Table 2:** Patient’s characteristics

Age (years)	47.8 ± 11.3
Sex (males)	214 (33.6%)
BMI (kg/m^2^)	50.2 ± 8.5
*Type of Surgery*	*Number of procedures (%)*
LRYGB	462 (72.6%)
LSG	128 (20.1%)
others	4 (0.7%)
Re-do operation	42 (6.6%)
LSG to LRYGB	22 (3.5%)
LSG to SADI-S	5 (0.8%)
LSG to mini-GB	3 (0.5%)
LSG to BPD-DS	3 (0.5%)
BPD-DS to LRYGB	1 (0.2%)
others	8 (1.3%)
*Co-morbidities*	*Number of patients (%)*
*DM type II*	
No	319 (50.2%)
Oral medication	208 (32.7%)
Insulin-therapy	109 (17.1%)
*GERD*	
No	455 (71.5%)
PPIs none/on demand	83 (13.1%)
PPIs regular	98 (15.4)
*OSAS*	
No	480 (75.5%)
Yes	156 (24.5%)
*NASH*	
No	407 (64%)
Yes	229 (36%)
*Liver cirrhosis*	
No	628 (98.7%)
Yes	8 (1.3%)

*BMI* Body Mass Index; *LRYGB* laparoscopic Roux-Y-gastric bypass; *LSG* laparoscopic sleeve gastrectomy; *SADI-S* single-anastomosis duodenoileal bypass with sleeve gastrectomy; *BPD-DS* biliopancreatic diversion with duodenal switch; *DM* diabetes mellitus; *GERD* gastroesophageal reflux disease; *OSAS* obstructive sleep apnea syndrome; *NASH* nonalcoholic steatohepatitis

### Endoscopic Findings

The most common abnormal endoscopic finding was gastritis (68.7%; *n* = 436). Urease test was positive in 111 patients (18.6%). Hiatal insufficiency or hiatal hernias were detected endoscopically in 207 patients (32.5%). Esophagitis was found in 139 patients (21.9%). Furthermore, esophagitis was detected in 8 patients, which had already undergone LSG (23.5%; *n* = 34). All other endoscopic abnormalities are summarized in Table 3.

**Table 3:** Endoscopic findings

*Abnormality*	*Number of patients (%)*
*Gastritis*	
No gastritis	199 (31.3%)
Erosive and/or hemorrhagic gastritis	94 (14.6%)
Mild chronic gastritis	343 (54.1%)
*Peptic ulcer*	
No peptic ulcer	612 (96.4%)
Gastric ulcer	17 (2.7%)
Duodenal ulcer	3 (0.5%)
Gastric and duodenal ulcer	2 (0.3%)
*Polyps*	
No polyps	593 (93.2%)
Gastric polyps	31 (4.9%)
Duodenal polyps	10 (1.6%)
Both	2 (0.3%)
*Gastric tumor*	
No tumor	626 (98.4%)
Submucosal tumor (e.g., GIST, lipoma)	9 (1.4%)
Gastric cancer	1 (0.2%)
*Hiatal hernia*	
No hernia	429 (67.5%)
Hiatal insufficiency or small hiatal hernia ≤ 3 cm	158 (24.8%)
Large hiatal hernia > 3 cm with/or without paraesophageal hernia	49 (7.7%)
*Esophagitis*	
No esophagitis	422 (66.4%)
Grade I	58 (9.1%)
Grade II	52 (8.2%)
Grade III	2 (0.3%)
Grade IV	1 (0.2%)
Barrett’s esophagus suspicious	75 (11.8%)
Z-line irregularity	24 (3.8%)
Candida esophagitis	2 (0.3%)
*Esophageal tumor*	
No tumor	625 (98.1%)
Submucosal tumor	7 (1.1%)
Distal esophageal cancer	3 (0.5%)

### Histological Findings

**Fig. 1:**
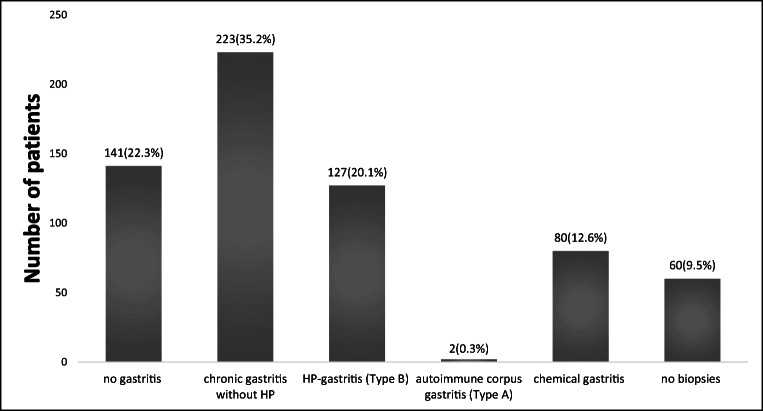
Classification of gastritis according to Sydney classification, histological division

Histologically, gastritis was found as the most frequent findings (68.2%; *n* = 432). The classification of gastritis is demonstrated in Fig. 1. There were 6 patients (1.0%) with gastric tumors: 4 benign tumors such as lipoma and leiomyoma, one patient with gastrointestinal stromal tumor (GIST), and one patient with gastric adenocarcinoma (cT1b, cN0, cM0, G3) and diffuse type according to Lauren’s criteria. Among 10 patients with intraoperatively detected GISTs, only one patient had been detected preoperatively via endoscopy [11]. The patient with the gastric-corpus carcinoma was confirmed preoperatively via endoscopy and histology. The planned procedure (LRYGB) was adapted into laparoscopic subtotal gastrectomy with D2-lymphadenectomy. Esophagitis was confirmed in almost all patients, who had macroscopic signs of manifested GERD in endoscopy (Table 4).

**Table 4:** Histological abnormalities of the biopsies collected via endoscopy in our cohort

*Findings*	*Number of patients (%)*	*Change of strategy*
*Gastritis*		
Non-atrophic gastritis with Hp infection	95 (15%)	Eradication therapy
Antrum restricted atrophic gastritis with Hp infection and IM (type B)	32 (5.1%)	Eradication therapy
Corpus limited atrophic gastritis with autoimmune origin (type A)	2 (0.3%)	Change of procedure from LRYGB to LSG
*Peptic ulcer*		
Gastric ulcer	17 (2.7%)	Eradication and re-endoscopy^1^
Duodenal ulcer or both	5 (0.8%)	
*Gastric tumors*		
Benign tumors	2 (0.3%)	Endosonography and excision
Polyps	1 (0.2%)	LRYGB to LSG
Gastric cancer	1 (0.2%)	LRYGB to subtotal gastrectomy + D2
*Esophagitis*	117 (18.4%)	LRYGB in 88 patients (75.2%)
*Barrett’s esophagus*	95 (15.0%)^2^	LRYGB in 72 patients (75.8%)
*Esophagus tumors*		
• Benign tumors	9 (1.4%)	Endosonography and excision
• Adenocarcinoma of GEJ (Siewert type I and II)	3 (0.5%)	Surgical resection(n = 2), definitive radio-chemotherapy(n = 1)^3^

*IM* intestinal metaplasia; *GEJ* gastroesophageal junction; ^1^the operation was postponed; ^2^one patient with low-grade dysplasia and one patient with high-grade dysplasia; ^3^surgical resection was not possible (BMI = 80 kg/m2)

Early esophageal cancer was detected endoscopically and confirmed by histology in three patients (cT1b, cN0, cM0). The scheduled procedure (LRYGB) was converted into laparoscopic extended transhiatal gastrectomy with D2 lymphadenectomy in two patients, whereas a surgical or even endoscopic resection was not feasible in the third patient, due to severe obesity (BMI > 80 kg/m^2^). In this patient, the protocol of definitive radiochemotherapy was carried out after MDT consensus.

### Univariate and Multivariate Analysis of Predictive Factors for Esophagitis and Barrett’s Esophagus in Bariatric Patients

The univariate analysis identified the following predictive factors as significant for esophagitis alone: History of GERD (*P* = 0.001), male gender (*P =* 0.02), and endoscopic hiatal hernia (*P* < 0.004). Other parameters, such as age, BMI, co-morbidities (DM type II, OSAS, liver cirrhosis), and history of re-do operation or LSG did not demonstrate any significant correlation with the endoscopic findings of esophagitis or BE (*P* > 0.1). In our cohort, BE was histologically confirmed in 95 patients (15.0%), the majority of whom were clinically asymptomatic (61.1%; *n* = 58/95) prior to endoscopy. The univariate analysis detected the following risk factors for the existence of BE, which reached the significance level of *P <* 0.05: History of GERD *P* = 0.04, hiatal hernia (endoscopic diagnosis) *P* < 0.001, and male gender *P* = 0.03. All parameters with a significance level *P* < 0.1 were included in a multivariate analysis in order to determine the predictive factors for esophagitis and BE. The multivariate analysis revealed the following significant risk factors for esophagitis: Medical history of GERD without regular therapy of PPIs, endoscopic detected hiatal hernia, and male gender, whereas only male gender and endoscopic detected hiatal hernia were predictive factors for BE (Table 5).

**Table 5:** Multivariate analysis of predictive factors for esophagitis or Barrett’s esophagus (preoperative model)

*Risk factors*	*Esophagitis*	*Barrett’s esophagus*
*GERD symptoms*	*P* = 0.002	*P* = 0.16
Without/on demand PPIs	*P* < 0.001	*P* = 0.26
	OR = 2.6	OR = 0.7
	CI = 1.6–4.4	CI = 0.4–1.3
With PPIs therapy	*P = 0.19*	*P* = 0.59
	OR = 1.4	OR = 1.2
	CI = 0.8–2.5	CI = 0.6–2.6
*Hiatal hernia*	*P* = 0.005	*P* < 0.001
Hiatal insufficiency or small ≤ 3 cm	*P* = 0.001	*P* < 0.001
	OR = 2.03	OR = 2.4
	CI = 1.3–3.1	CI = 2.5–4.03
Large > 3 cm	*P = 0.5*	*P* = 0.002
	OR = 1.3	OR = 3.1
	CI = 0.6–2.8	CI = 1.5–6.5
*Gender (male)*	*P* = 0.004	*P* = 0.08
	OR = 1.9	OR = 1.9
	CI = 1.2–2.7	CI = 1.2–2

### Impact on Operative Strategy

**Fig. 2:**
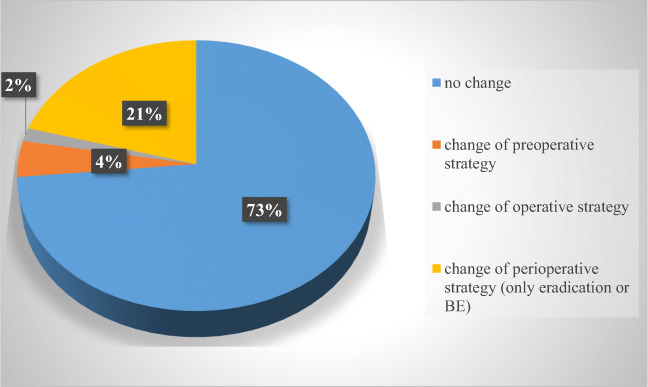
The impact of preoperative endoscopy on our peri-operative strategy

The data presented here based on the preoperative endoscopic and histologic findings showed that the preoperative upper-GI endoscopy resulted in no change of bariatric surgical therapy in 460 patients (72.3%), whereas it leads to a significant change of perioperative workup in 166 patients (26.1%), while a change of the final operative strategy occurred in 10 patients (1.6%) only. Detailed results about the change of the peri−/operative strategy are demonstrated in Fig. 2.

A hiatal hernia was described in 207 Cases (32.5%) preoperatively. However, a relevant hernia was found intraoperatively only in 96 Cases (15.1%).

## Discussion

The value of preoperative upper-GI endoscopy prior to bariatric surgery is still in debate nowadays. Several previous reports have emphasized its benefits in the exclusion of clinically relevant upper GI-tract pathologies, which facilitates a more holistic patient’s management and a rational selection of the best surgical approach in order to maximize the bariatric effect (Table 6). The recently published German bariatric surgery guidelines focus on the crucial part of preoperative upper-GI endoscopy in the perioperative workup of obese patients and recommend it prior to each bariatric surgery [6, 19]. In contrast, other reports have underlined that upper-GI endoscopy has impaired impact on the bariatric procedure itself and thus should be considered in symptomatic patients only [12, 15, 20]. Consequently, the ASMBS recommends that the decision to perform endoscopy prior to bariatric surgery should be individualized depending on the type of the operation [7]. Currently, the European Chapter of the International Federation for the Surgery of Obesity EC-IFSO has no formal recommendation [21]. The data and recommendations of the surgical and gastrointestinal societies are variable and potentially conflicting and have not reached a high evidence level yet (Table 6). In this study, we assessed the role of upper-GI endoscopy in bariatric surgery and focused the question, how it might lead to changes in the perioperative management. Probably, endoscopy is more relevant in centers which perform LRYGB as the main procedure, and the remnant stomach is no longer accessible endoscopically. Furthermore, the majority of reports, which discourage the routine upper-GI endoscopy, are based on patients, who had not undergone LRYGB [12, 22].

**Table 6:** Most studies addressing routine gastroscopy prior to bariatric surgery with variable results and recommendations

*Study/procedure (year)*	*Number and race of patients*	*Abnormal findings (%)*	*Change of surgical approach (%)*	*Recommendation of routine EGD*
Colman RJ, et al. [12] LSG (2019)	94, adolescents	(46%) hiatal hernia 4.2% Abnormalities of gastric mucosa 38.3%	no	no
D’Silva M, et al. [4] LRYGB & LSG (2018)	675 Indians	(79%) hiatal hernia ≈52.5%, esophagitis ≈17%, BE ≈2 gastritis ≈46% polyps ≈2.5% (incl. 2 GIST, 6 leiomyomas, 6 NETs)	yes (9.93%)	yes
Lee J, et al. [13] LRYGB & LSG (2017)	268 Asians	(51%) hiatal hernia ≈18%, esophagitis ≈7.5% gastritis ≈32,5%, H.p. ≈24% no malignancies	yes (0.7%)	yes
Wolter S, et al. [14] LRYGB & LSG (2017)	801 Caucasians	(65.7%) hiatal hernia 22%, GERD ≈25%, BE ≈2% gastritis ≈32%, gastric erosions ≈2% malignancies 0,5% (incl. 1 GIST, 1 NET, 2 adenocarcinomas)	data n/a	yes
Abd Ellatif ME, et al. [15] LSG, LRYGB & MGB (2016)	3219 Arabics	(25%) hiatal hernia ≈30%, gastritis 23%, esophagitis 15%, ulcers ≈3%, BE 1.2%. Benign polyps 0.12%	no	no
Ng JY, et al. [16] LRYGB & LSG (2016)	208 Asians	(≈66%)hiatal hernia ≈16%, esophagitis ≈2% gastritis ≈50%, erosive gastritis ≈5%, H.p. ≈14% peptic ulcer ≈5%, Malignancies ≈0,5%	yes (≈ 5.2%)	yes
Schigt A, et al. [17] LRYGB (2014)	523 Caucasians	(≈17%), no data a/v	no	no
Peromaa-Haavisto P, et al. [18] LRYGGB, (2013)	412 Caucasians	(54%) hiatal hernie 25.4%, esophagitis13.2%, BE 1.2% gastritis ≈13.7%, H.p. 12%, ulcers ≈2.9% benign polyps 6.7% (1 Leiomyoma) no malignant lesions	no	no

*EGD* Esophagogastroduodenoscopy; *LSG* laparoscopic sleeve gastrectomy; *LRYGB* laparoscopic Roux-Y-gastric bypass; *BE* Barrett’s esophagus; *GIST* gastrointestinal tumor; *NET* neuroendocrine tumor; *H.p.* Helicobacter pylori

Mild chronic gastritis was the most common endoscopic finding in our study. A meta-analysis of 18 studies has also demonstrated moderate gastritis as the most frequent pathological finding in bariatric patients as well [23]. We discussed our results further according to its potential influence on the perioperative management.

### Hp-Gastritis and Peptic Ulcer

*H. pylori* is one of the most common causes that has been linked to active chronic gastritis, gastric ulcer, and gastric cancer [24]. The incidence rate of Hp infection in Western Europe including Germany is declining. Hooi et al. reported in a systematic review of population-based studies an Hp prevalence of 35% in the German general population [25], which is almost similar to our results (22.5%). Hp infection and gastric ulcer have been described as a risk factor in patients undergoing LRYGB for developing marginal ulcer (MU) postoperatively [26]. Furthermore, eradication therapy and prophylactic PPIs-therapy might be useful to prevent MU postoperatively [26]. Principally, there are different indirect ways to confirm Hp infection such as serology, urea breath test (UBT), and stool antigen test (SAT), but these tests do not allow us to evaluate other pathological finding such as gastric ulcer, atrophic gastritis, and gastric cancer preoperatively. This might be an issue, especially in patients undergoing LRYGB, where the gastric remnant will no longer be easily accessible. Furthermore, it might be useful to prove the effectivity of the eradication therapy in patients with gastric ulcer prior to excluding the stomach through LRYGB. However, there are data regarding any patient’s benefit, even the need of eradication of asymptomatic patients undergoing obesity surgery is discussed with controversy. However, Fernandes et al. have already described gastric ulcer as a predictor for postoperative complications in patients who underwent bariatric surgery [27]. In our center, a standard eradication therapy was performed in patients with Hp gastritis (22.5%; *n* = 143). We had 22 patients (3.5%) with peptic ulcer. Strategy of therapy was altered, and the operation was delayed until the ulcer had healed (controlled by upper-GI endoscopy with new biopsies to exclude malignancy). Furthermore, the operative strategy was also altered (from LRYGB to LSG) in two patients with autoimmune gastritis (AIG) as a risk factor for gastric cancer, in order to be able to control the stomach easier after bariatric surgery [28]. In this study with retrospective nature, we could not assess objectively the symptoms of patients with gastric ulcer, as the documents of the electronic patient chart did not reveal clear symptoms in the majority of the patients. However, the usefulness of these symptoms as an indication for performing upper-GI endoscopy in bariatric surgery is still under controversy [29].

### GERD and Barrett’s Esophagus

Obesity is as an independent risk factor for GERD [30, 31]. The presence of GERD in patients undergoing bariatric surgery has been reported to be 30–60% [32], which is almost similar to our results (29%). Furthermore, various guidelines and reports have already described GERD as an obesity-related disease [19, 21]. Consequently, a relationship of obesity with GERD-related disorders such as esophagitis, BE, and malignancies has been also reported in many studies. A meta-analysis by El-Serag et al. had shown a significant association between obesity and esophagitis [31]. In a systematic review by Seidel et al., obesity was presented as a risk factor for developing Barrett’s esophagus [3]. We have also defined GERD without regular PPIs treatment as risk factor for esophagitis (Table 5). In our report, we confirmed esophagitis in 139 patients (21.9%) by endoscopic biopsies and Barrett’s esophagus in 95 patients (15.0%), which are higher incidence rates compared to other studies (Table 6).

There are many reports about the risk of increasing GERD symptoms or developing real “de novoˮ GERD after LSG [33]. Furthermore, Felsenreich et al. described a comparable high incidence (15%) of Barrett’s esophagus after LSG at 10 years postoperatively [34]. However, we could demonstrate that the reason to perform the re-do operations in about half of our patients with LSG was GERD (58.3%, 21/36) with a significant association (*P* < 0.001). Furthermore, many reports described LRYGB as a gold standard in obese patients with GERD, demonstrating an effective relief of symptoms and regression of Barrett’s mucosa [35, 36]. Thus, we avoid performing LSG in patients with GERD, reflux esophagitis, or BE. In case of extremely obese patients where LRYGB is technically not possible and LSG is performed (two-step concept), patients will receive a follow-up upper-GI endoscopy after 1 year, and eventually they are scheduled for a re-operation and converting the LSG into LRYGB. According to the recent guidelines, all patients with new diagnosed BE without dysplasia in our center receive an endoscopic control after 1 year and then every 3–5 years [10, 37]. In a Finnish cohort, BE has been described as a rare disease (1%) and the patients with Barrett’s esophagus without dysplasia were not further followed-up endoscopy [18]. Thus, a routine upper-GI endoscopy was discouraged before LRYGB. We cannot support this concept, as there is a big difference with the data from our cohort. Patients with BE still need an endoscopic surveillance with thorough evaluation of further mucosal transformation toward malignancy [10, 37]. In our study, we found that the existence of hiatal hernia and the male gender are main risk factors for BE, although the majority of patients with BE were asymptomatic (61.1%). Thus, we cannot support the recommendation of performing upper-GI endoscopy in symptomatic patients only. Taking together the incoherency of the different study groups from all around the world regarding the incidence of Barrett’s esophagus, one might suppose a regional variability [38]. This variability might be due to different dietary habits.

### Benign and Malignant Tumors

In our cohort analysis, four patients were found with upper-GI malignancies (0.7%). Several reports support the association between obesity and development of esophageal adenocarcinoma. Ryan et al. described that the incidence of adenocarcinoma in the distal esophagus and gastric cardia was 11.3 times higher in obese patients (BMI > 30 kg/m^2^) compared to normal patients (BMI < 22 kg/m^2)^ [39]. Thus, obese patients undergoing bariatric surgery are automatically a high-risk group for esophageal adenocarcinoma. Therefore, in these patients, a routine upper-GI endoscopy appears highly relevant from an oncological point of view, in order to detect the cancer as early as possible at a curative stage.

## Conclusion

In this study, we document a relatively high incidence of a wide range of abnormalities in upper-GI tract of obese patients, which can be detected by routine upper-GI endoscopy prior to the bariatric procedure. Pathologic findings, such as peptic ulcer, esophagitis, Barrett’s esophagus, and esophageal cancer remain largely asymptomatic but may become highly relevant with regard to patients’ morbidity and mortality following bariatric procedures. Additionally, upper-GI endoscopy does not only influence the surgical pathway but also the bariatric follow-up, and in conjunction with the very low complication rates associated with the endoscopic procedure, it should be routinely performed prior bariatric surgery.

## Footnotes

1 Patients were divided according to their BMI into 4 groups: group1 ≥ 30–40, group 2 ≥ 40–50, group 3 ≥ 50–60, and group 4 ≥ 60 kg/m^2^
