# Correction to: Preoperative Upper-GI Endoscopy Prior to Bariatric Surgery: Essential or Optional?

**DOI:** 10.1007/s11695-021-05765-4

**Published:** 2021-11-03

**Authors:** Yusef Moulla, Orestis Lyros, Matthias Mehdorn, Undine Lange, Haitham Hamade, Rene Thieme, Albrecht Hoffmeister, Jürgen Feisthammel, Matthias Blüher, Boris Jansen-Winkeln, Ines Gockel, Arne Dietrich

**Affiliations:** 1 Department of Abdominal, Transplant, Thoracic and Vascular Surgery, University Hospital of Leipzig, Liebigstrasse 20, D-04103 Leipzig, Germany; 2 Integrated Research and Treatment Center (IFB) Adiposity Diseases, Leipzig, Germany; 3 Clinic for Gastroenterology, Department of Internal Medicine, University Hospital of Leipzig, Liebigstrasse 20, D-04103 Leipzig, Germany

**Correction to: Obesity Surgery (2020) 30:2076–2084**

**https://doi.org/10.1007/s11695-020-04485-5**

The article “Preoperative Upper-GI Endoscopy Prior to Bariatric Surgery: Essential or Optional?” by Yusef Moulla, Orestis Lyros, Matthias Mehdorn, Undine Lange, Haitham Hamade, Rene Thieme, Albrecht Hoffmeister, Jürgen Feisthammel, Matthias Blüher, Boris Jansen-Winkeln, Ines Gockel, and Arne Dietrich was published online on February 24, 2020 with the copyright holder “Springer Nature”. With the decision to publish with Open Access the copyright holder has changed to “The Author(s)”. The original article has been revised to change the copyright holder and to add the Open Access funding note.

